# Author Correction: Nano-imaging photoresponse in a moiré unit cell of minimally twisted bilayer graphene

**DOI:** 10.1038/s41467-022-29014-z

**Published:** 2022-03-07

**Authors:** Niels C. H. Hesp, Iacopo Torre, David Barcons-Ruiz, Hanan Herzig Sheinfux, Kenji Watanabe, Takashi Taniguchi, Roshan Krishna Kumar, Frank H. L. Koppens

**Affiliations:** 1 ICFO-Institut de Ciencies Fotoniques, The Barcelona Institute of Science and Technology, Barcelona, Spain; 2 Research Center for Functional Materials, National Institute for Materials Science, Tsukuba, Ibaraki Japan; 3 International Center for Materials Nanoarchitectonics, National Institute for Materials Science, Tsukuba, Ibaraki Japan; 4 ICREA-Institució Catalana de Recerca i Estudis Avançats, Barcelona, Spain

**Keywords:** Optical properties and devices, Near-infrared spectroscopy, Electronic properties and materials

Correction to: *Nature Communications* 10.1038/s41467-021-21862-5, published online 12 March 2021.

The original version of this Article omitted the following from the Acknowledgements: “F.H.L.K. also acknowledges support from the PID2019-106875GB-I00 project funded by MCIN/ AEI /10.13039/501100011033”. This has now been corrected in both the PDF and HTML versions of the Article.

