# Supplementary material for: Nano-imaging photoresponse in a moiré unit cell of minimally twisted bilayer graphene
Source: Nat Commun. 2021 Mar 12;12:1640. doi: 10.1038/s41467-021-21862-5 (PMC7954806; doi:10.1038/s41467-021-21862-5)
Supplement: Supplementary file 1 — Supplementary Information [file 41467_2021_21862_MOESM1_ESM.pdf]

Supplementary Information

Nano-imaging photoresponse in a moiré unit cell of minimally twisted bilayer graphene

Niels C.H. Hesp[1], Iacopo Torre[1], David Barcons Ruiz[1], Hanan Herzig Sheinfux[1], Kenji Watanabe[2], Takashi Taniguchi[3], Roshan Krishna Kumar[1]*, Frank H.L. Koppens[1,4]*

[1]ICFO-Institut de Ciencies Fotoniques, The Barcelona Institute of Science and Technology, 08860 Castelldefels (Barcelona), Spain.

[2]Research Center for Functional Materials, National Institute for Materials Science, Namiki 1-1, Tsukuba, Ibaraki 305-0044, Japan.

[3]International Center for Materials Nanoarchitectonics, National Institute for Materials Science, Namiki 1-1, Tsukuba, Ibaraki 305-0044, Japan.

[4]ICREA-Institució Catalana de Recerca i Estudis Avançats, 08010 Barcelona, Spain.

*To whom correspondence should be addressed: roshan.krishnakumar@icfo.eu, frank.koppens@icfo.eu

This file contains the following Supplementary Notes:

1. Carrier density calibration

2. Photoresponse in other mTBG devices

3. Estimation of charge neutrality point

4. Photo-thermoelectric effect

5. Photo-thermoelectric effect in a 1D channel

6. Calculation of thermoelectric transport coefficients in mTBG

7. Impact of strain inside the AB domains

8. Cooling length in our devices

9. Effects beyond the photo-thermoelectric effect

10. Heating from hyperbolic phonon polaritons

Supplementary references

Supplementary Note 1: Carrier density calibration

In field-effect transistor geometries, the carrier density (n) induced by an applied gate voltage is generally well described by a simple capacitance model. Even so, Hall-effect measurements are usually a pre-requisite in proper characterisation of n in any conducting system and allows one to make a calibration of the n induced by the applied gate voltage (V_G). In our devices, however, measurement geometries are not-well suited for Hall measurements. To accurately determine the induced n in our devices, we instead perform a calibration by measuring the plasmon-polariton dispersion in a single-layer graphene (SLG) region. Infrared plasmon-polaritons have been studied extensively in graphene, and their dispersions are well known[1–3]. By studying the near-field photocurrent close to a PN junction, we directly image graphene plasmons, measure their wavelength, and determine the doping level n that such an excitation corresponds to. Moreover, tuning the gate-voltage tunes the plasmon wavelength, which allows us to make a calibration of $n(V_G)$. Supplementary Fig. 1a plots a map of the measured photovoltage (we plot the derivative with respect to x_{tip} to make features clearer) as a function of gate voltage and tip position (x_{tip}), where the x-axis corresponds to a spatial line scan made near one of the Au measurement contacts of our device (purple dot in Fig. 1b of the main text). The Au-SLG interface is marked by the dashed line in Supplementary Fig. 1a. In line with the photo-thermoelectric effect, the photovoltage changes sign when the gate is tuned through the charge neutrality point of graphene. On top of this, we can also see a set of fringes that become wider spaced at higher gate voltages. They arise from thermoelectric detection[2,3] of the interfering plasmon-polaritons in graphene. To extract the plasmon wavelengths, we follow the method described in Ref. 1, that involves fitting a polynomial combined with a sinusoidal function (see inset of Supplementary Fig. 1b). The corresponding n for the measured plasmon wavelength is then determined from the plasmon dispersion relation (Supplementary Fig. 1b) calculated in our sample at the excitation energy used in our measurement (117 meV)[1]. With this method, we obtain the density calibration plotted in Supplementary Fig. 1c. It shows a linear behaviour with gate-voltage as expected. Note, n is slightly higher than what is typically expected for the dielectric thickness of our capacitor, which we attribute to photodoping[4] caused by the constant far-field infrared illumination that is unavoidable in our SNOM experiments.

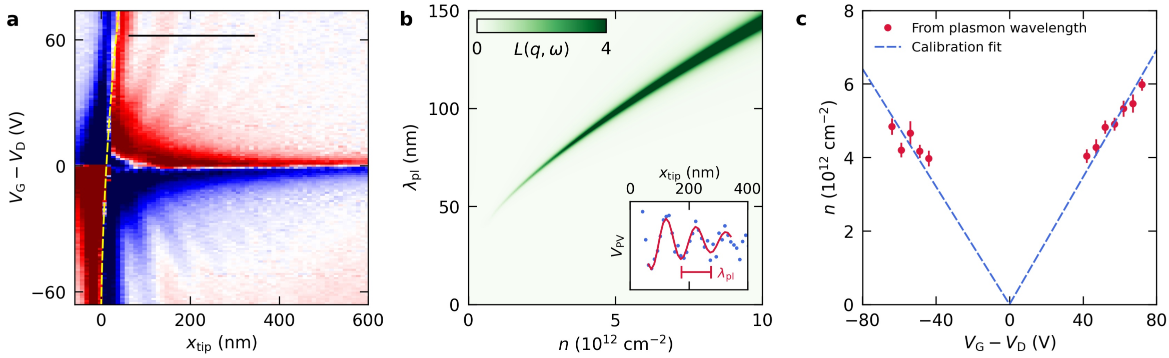

Supplementary Figure 1 | Estimation of the induced carrier density for an applied gate voltage. a Derivative along x_{tip} of the measured photovoltage in single-layer graphene near a contact for a range of gate voltages. The contact (extending up to the yellow dashed line) serves as a launcher for plasmon-polaritons in SLG, observed as periodic oscillations in the plot. The excitation energy is 117 meV. **b** Calculated loss function of SLG in our sample at an excitation energy of 117 meV for various carrier densities. The sharp green line corresponds to the plasmon-polariton resonance, which changes its wavelength λ_{pl} with carrier density. Inset: measured photovoltage along the black trace in panel **a**, together with a fit (red line) to extract λ_{pl}. **c** Extracted carrier density for various gate voltages based on the data of panel **a** and the calculated dispersion relation shown in panel **b**. The blue dashed lines are linear fits on either side of the CNP, giving an estimation of the induced carrier density at lower gate voltages. The errorbars represent the ±1σ standard deviation.

Supplementary Note 2: Photoresponse in other mTBG devices

The behaviour reported in the main text was found to be generic to our other measured mTBG devices. To illustrate this, we plot measured photocurrent maps of another mTBG device (Supplementary Fig. 2a). Although slightly more anisotropic, the triangular patterns of the moiré lattice intrinsic to mTBG can be seen and are similar to those measured in the contact configuration used in Fig. 2a of the main text. We find the same qualitative behaviour including sign changes across certain domain walls, in line with what is expected from the PTE (Fig. 3a-c of the main text), and the double-step feature at domain wall interfaces (see dotted lines in Supplementary Fig. 2a).

The measurements presented in the main text were focused on mTBG with moiré structures of size ~500 nm. However, we also observed structures with smaller periodicities. Supplementary Fig. 2b plots the measured photocurrent of the device presented in the main text, but in the mTBG region on the bottom side of the single-layer graphene region. Again, we find the same periodic patterns in the photocurrent as observed in larger structures. Whilst the seemingly square lattice we observe in Supplementary Fig. 2b is not representative of the moiré lattice in mTBG, we recover the triangular lattice (see shaded domains) by incorporating the same directional effect as to that reported in the main text (Fig. 2a of the main text).

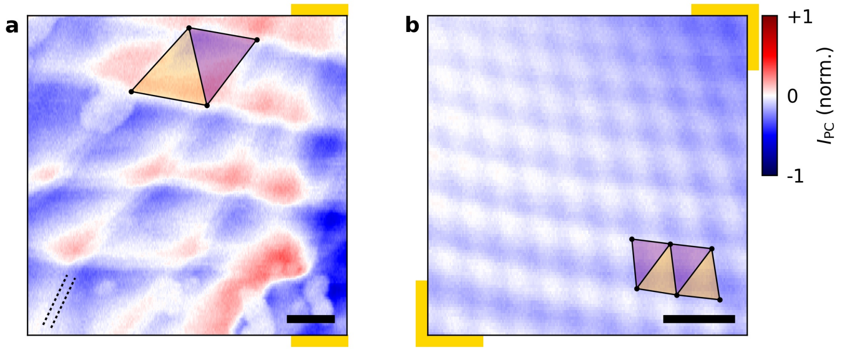

Supplementary Figure 2 | Near-field photocurrent images in other regions/devices. a Photocurrent image taken in one of our other devices at E = 117 meV near charge-neutrality. The profile is qualitatively the same as in Fig. 2**a** of the main text, due to a similar arrangement of domain walls with respect to the current path lines between measurement contacts. **b** Photocurrent image of a higher-density network with moiré domains as small as ~100 nm, measured at E = 188 meV near charge-neutrality. In both panels the yellow/purple triangles indicate the location of AB/BA domains, and the gold patches give a rough indication of the source/drain contacts. Length of scale bar is 200 nm in both panels.

Supplementary Note 3: Estimation of charge neutrality point

To assess the position of the charge neutrality point (CNP) in our gate scan of the photocurrent (Fig. 2d of the main text), we simultaneously measured the near-field optical scattering signal. The scattered signal essentially measures the Drude response in our samples and changes smoothly with doping, showing a minimum at CNP[1]. This method enables local probing of the chemical potential and allows us to determine the gate voltage (V_G) that corresponds to the CNP. Supplementary Fig. 3 plots the photocurrent traces (left axis) as already displayed in Fig. 2d of the main text, along with the phase of the optically scattered signal (red dashed line, right axis). The minimum phase shift occurs at V_G = 10.5 V, signals the position of the CNP.

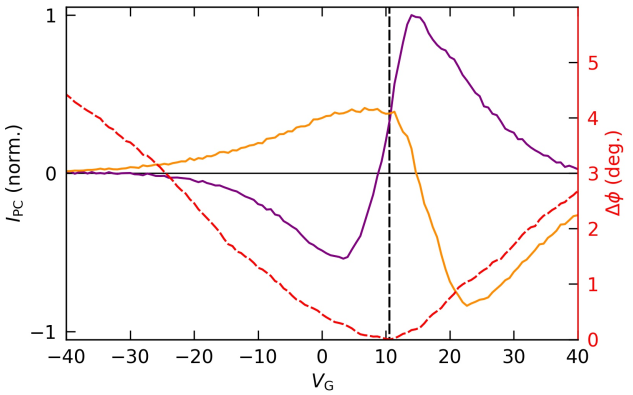

Supplementary Figure 3 | Charge neutrality point measured by s-SNOM. The dashed red trace (right axis) corresponds to the scattered optical phase recorded during the gate scan in Fig. 2**d** of the main text, providing a local probe of the CNP. As a comparison, we plot the same photocurrent traces shows in Fig. 2**d** (left axis).

Supplementary Note 4: Photo-thermoelectric effect

Our description of the photo-thermoelectric effect (PTE) is based on two local linear response equations. The first reads

$$\mathbf{J}(\mathbf{r}) = -\sigma(\mathbf{r})\nabla V(\mathbf{r}) - \sigma(\mathbf{r})S(\mathbf{r})\nabla\delta T(\mathbf{r}), \quad \text{(S1)}$$

where $\mathbf{J}(\mathbf{r})$ is the electric current density, $\sigma(\mathbf{r})$ is the local, direct-current (DC) conductivity, $V(\mathbf{r})$ the electric potential, $S(\mathbf{r})$ the Seebeck coefficient, and $\delta T(\mathbf{r}) \equiv T(\mathbf{r}) - T_0$ is the temperature deviation from the substrate temperature T_0, which we assume to be constant. The first term is simply the local form of Ohms law, while the second represents the Seebeck effect, i.e. an electric current driven by a temperature gradient.

The second equation involves the heat current density $\mathbf{q}(\mathbf{r})$ and is given by

$$\mathbf{q}(\mathbf{r}) = -\kappa(\mathbf{r})\nabla\delta T(\mathbf{r}) + \Pi(\mathbf{r})\mathbf{J}(\mathbf{r}), \quad \text{(S2)}$$

where $\kappa(\mathbf{r})$ is the thermal conductivity and $\Pi(\mathbf{r}) = T(\mathbf{r})S(\mathbf{r}) \approx T_0 S(\mathbf{r})$ is the Peltier coefficient. The first term describes the normal heat conduction (Fourier law) while the second describes the heat current generated by a flowing electric current, known as the Peltier effect.

At steady state, the following continuity equations for the two currents hold

$$\nabla \cdot \mathbf{J}(\mathbf{r}) = 0, \quad \text{(S3)}$$

$$\nabla \cdot \mathbf{q}(\mathbf{r}) = -g(\mathbf{r})\delta T(\mathbf{r}) + Q(\mathbf{r}). \quad \text{(S4)}$$

The first equation simply expresses charge conservation, while the second represents heat dissipation to the substrate (first term, $g(\mathbf{r})$ being the thermal coupling to the substrate) or heat generation by light absorption (second term) as described in the main text.

Taking the divergence of (S2) and using (S3) and (S4) yields the temperature diffusion equation

$$-\nabla[\kappa(\mathbf{r})\nabla\delta T(\mathbf{r})] + g(\mathbf{r})\delta T(\mathbf{r}) = Q(\mathbf{r}) - T_0\nabla S(\mathbf{r}) \cdot \mathbf{J}(\mathbf{r}), \quad \text{(S5)}$$

where we can distinguish two source terms: the external heat introduced into the system $Q(\mathbf{r})$ and the heat generated by a flow of electric current via the Peltier effect. Note that the heat generated through the Joule effect by the steady current $\mathbf{J}(\mathbf{r})$ is neglected since it is quadratic in $\mathbf{J}(\mathbf{r})$, and hence quadratic in the absorbed power. If we assume that both κ and g are spatially homogeneous, Eq. (S5) simplifies to

$$-\nabla^2\delta T(\mathbf{r}) + L_{\mathrm{cool}}^{-2}\delta T(\mathbf{r}) = \frac{1}{\kappa}[Q(\mathbf{r}) - T_0\nabla S(\mathbf{r}) \cdot \mathbf{J}(\mathbf{r})], \quad \text{(S6)}$$

where $L_{\mathrm{cool}} \equiv \sqrt{\kappa/g}$ is the cooling length that determines how far the heat can travel in the sample before being lost to the substrate because of out-of-plane conduction.

Equations (S1-4) can be combined into a linear system of Partial Differential Equations (PDEs) in the form

$$-\nabla \cdot \left[\begin{pmatrix} \sigma(\mathbf{r}) & T_0\sigma(\mathbf{r})S(\mathbf{r}) \\ T_0\sigma(\mathbf{r})S(\mathbf{r}) & T_0^2\sigma(\mathbf{r})S^2(\mathbf{r}) + T_0\kappa(\mathbf{r}) \end{pmatrix}\begin{pmatrix} \nabla V(\mathbf{r}) \\ T_0^{-1}\nabla\delta T(\mathbf{r}) \end{pmatrix}\right] + \begin{pmatrix} 0 \\ g(\mathbf{r})\delta T(\mathbf{r}) \end{pmatrix} = \begin{pmatrix} 0 \\ Q(\mathbf{r}) \end{pmatrix}. \quad \text{(S7)}$$

Here we put the equations in a form that makes explicit the symmetry of the coefficient matrix due to Onsager relations.

At the m-th contact the voltage has a constant value $V(\mathbf{r}) = V_m$, while the current flowing in it is given as $I_m = \int_{\text{Contact } m} \mathbf{J}(\mathbf{r}) \cdot \hat{\mathbf{n}}\, ds$, $\hat{\mathbf{n}}$ being the outward normal unit vector (we consider positive currents those leaving the device).

In our experiment $V_1 = 0$ and $I_2, I_3 = 0$ (see Fig. 3d of the main text for contact numbering). These conditions, together with the boundary conditions on the temperature field $\delta T(\mathbf{r}) = 0$ in the contacts, and $\mathbf{q}(\mathbf{r}) \cdot \hat{\mathbf{n}} = 0$ on the rest of the boundary, specify uniquely the solution[5] of the problem (S7) given the heat source $Q(\mathbf{r})$.

Solving (S7) numerically via finite element method (FEM) allows calculating $V_2[Q]$ and $V_3[Q]$ from the solution. Because of the linearity of the problem it is in principle possible to solve the system (S7) for a point source located at $\mathbf{r}_0$, i.e. $Q_{\text{point}}(\mathbf{r}, \mathbf{r}_0) = \delta(\mathbf{r} - \mathbf{r}_0)$ and obtain the results for a generic source in the form

$$V_{2/3}[Q] = \int d\mathbf{r}_0\, \mathcal{R}_{\text{PTE}}^{(2/3)}(\mathbf{r}_0) Q(\mathbf{r}_0), \tag{S8}$$

where the photovoltage responsivities $\mathcal{R}_{\text{PTE}}^{(m)}(\mathbf{r}_0)$ are obtained by evaluating V_m on the solution corresponding to the point source $Q_{\text{point}}(\mathbf{r}, \mathbf{r}_0)$. This approach is numerically intense, since it requires calculating the solution of (S7) one time for every position at which we want to know the responsivities. We can instead make use of the elegant reciprocity principle[5,6] to solve for the responsivities in one shot.

This reciprocity principle affirms, for our experimental configuration, that $\mathcal{R}_{\text{PTE}}^{(2)}(\mathbf{r}_0)$ is equal to the normalized temperature field $\delta T(\mathbf{r})/(I_0 T_0)$ obtained by solving (S7) with $V_1 = 0, I_2 = I_0$, and $I_3 = 0$, while $\mathcal{R}_{\text{PTE}}^{(3)}(\mathbf{r}_0)$ is equal to the temperature field obtained by solving (S7) with $V_1 = 0, I_2 = 0$, and $I_3 = I_0$. Solving these two PDE problems with the FEM code[5] we obtained the responsivity maps shown in the main text. We note that this picture is modified in presence of resonant response (either in the sample or the substrate). In this case $Q(\mathbf{r}, \mathbf{r}_{\text{tip}})$ can spread considerably away from the tip[2] giving rise to additional features.

In the following, we describe the details of the parameters that we feed into the simulations. First, based on sample characterization via AFM/s-SNOM/Raman/photocurrent measurements, we define the sample geometry with specific regions consisting of SLG, and other regions of mTBG (see Fig. 3d of the main text). Next, using the scattering data as a guide (Fig. 1c of the main text), we draw the network of domains in the region of interest, as reflected by Fig. 3e of the main text. Using this geometry, we generate a sample mesh with a variable cell size, with those closest to the domain walls having the smallest edge size of about 1.5 nm.

We define at each cell of the mesh the input parameters as follows:

- In mTBG we consider the domain wall to be of the shear-type[7]. This means that we should take the xx-component of the conductivity and Seebeck tensors[8] as defined in the Supplementary Note 6. We evaluate the Seebeck coefficient S_{xx} and and DC conductivity $\sigma_{xx}(\omega = 0)$ at $T_0 = 300\ \text{K}$ with an energy broadening $\eta = 10\ \text{meV}$ corresponding to a scattering time of ~ 400 fs.
- In SLG, we use the Mott formula $S(\mu) = -\frac{\pi^2 k_{\text{B}}^2 T}{3e} \frac{1}{\sigma} \frac{\mathrm{d}\sigma}{\mathrm{d}\mu}$, with $\sigma(n) \propto 1 + \frac{n(\mu)}{n^*}$ as the DC conductivity, $\mu = \hbar v \sqrt{\pi n}$, and $n^* = 8 \cdot 10^{10}\ \text{cm}^{-2}$ as the impurity density determined for our device – in agreement with what is expected for graphene on hexagonal-boron nitride[9]. To simulate accurately the influence of SLG in the potential landscape, the conductivity serving

as input for the simulation is set to 1.5x the value of AB-stacked BLG, which is a typical for hBN-encapsulated SLG/BLG devices[10].

- The thermal conductivity κ is given at each point using the Wiedemann-Franz law, $\kappa = \frac{\pi^2 k_B^2 T}{3e^2}\sigma$, using the electronic conductivities as defined above.
- The thermal coupling to the substrate is in large part governed via coupling of hot electrons to hBN phonons[11] with a coupling coefficient $g \approx 5 \cdot 10^4\ \mathrm{WK^{-1}m^{-2}}$. We take this value for the mTBG and SLG regions, leading to a cooling length $L_{\mathrm{cool}} \approx 270\ \mathrm{nm}$ in mTBG. This compares well with the experimental cooling length extracted in mTBG (see Supplementary Note 8).

Supplementary Note 5: Photo-thermoelectric effect in a 1D channel

A better grasp of the qualitative features of the responsivities can be obtained by solving the thermoelectric equations in a one-dimensional (1D) setting, where calculations can be carried out analytically. We consider a 1D channel wherein all the variables depend only on the longitudinal (x) coordinate. By symmetry, all the vector fields point in the $\hat{x}$ direction. As a further simplification we consider σ, κ and g as constants, independent on the position. Moreover, we assume that two contacts are placed at $\pm L/2$, with $L \gg L_{cool} = \sqrt{\kappa/g}$, and that heat sources $Q(x)$ and Seebeck coefficient gradients $\partial_x S(x)$ are localized far from the contacts. The continuity Equation (S3) can be directly integrated, yielding

$$j_x(x) = \frac{I}{W}, \tag{S9}$$

The potential difference between the two contacts can be obtained by integrating the electric current Equation (S1) and reads

$$\Delta V \equiv V\left(-\frac{L}{2}\right) - V\left(\frac{L}{2}\right) = IR + \int_{-\frac{L}{2}}^{\frac{L}{2}} dx' S(x') \partial_x \delta T(x') = IR - \int_{-\frac{L}{2}}^{\frac{L}{2}} dx' \partial_x S(x') \delta T(x') . \tag{S10}$$

Here, $R = L/(W\sigma)$ is the resistance and we integrated by parts the last term imposing the boundary condition $\delta T(x) = 0$ for $x = \pm L/2$. The heat conduction Equation (S6) can be inverted using the Green function of the 1D diffusion equation that is decaying far from the origin

$$[-\partial_x^2 + L_{\rm cool}^{-2}] \frac{L_{\rm cool}}{2} e^{-\frac{|x|}{L_{\rm cool}}} = \delta(x) , \tag{S11}$$

yielding

$$\delta T(x') = \int_{-L/2}^{L/2} dx'' \frac{L_{\rm cool}}{2} e^{-\frac{|x'-x''|}{L_{\rm cool}}} \frac{1}{\kappa} \left[Q(x'') - \frac{I T_0}{W} \partial_x S(x'') \right]. \tag{S12}$$

In an open-circuit configuration ($I = 0$) the above equations simplify and we can write the photovoltage, substituting (S12) into (S10) as

$$\Delta V = W \int_{-\frac{L}{2}}^{\frac{L}{2}} dx'' \, \mathcal{R}(x'') Q(x''), \tag{S13}$$

with the photovoltage responsivity given by

$$\mathcal{R}(x'') = -\frac{1}{\kappa W} \int_{-\frac{L}{2}}^{\frac{L}{2}} dx' \frac{L_{\rm cool}}{2} e^{-\frac{|x'-x''|}{L_{\rm cool}}} \partial_x S(x') . \tag{S14}$$

Note that, according to (S12) the normalized temperature profile in absence of heat sources but with $I = I_0$ reads

$$\frac{\delta T(x')}{I_0 T_0} = -\frac{1}{\kappa W} \int_{-\frac{L}{2}}^{\frac{L}{2}} dx'' \frac{L_{\rm cool}}{2} e^{-\frac{|x'-x''|}{L_{\rm cool}}} \partial_x S(x''), \tag{S15}$$

thus explicitly confirming the validity of the reciprocity approach in this simplified 1D setting.

Supplementary Note 6: Calculation of thermoelectric transport coefficients in mTBG

The strategy to calculate the relevant thermodynamic quantities of mTBG in the vicinity of domain walls, including the DC conductivity tensor $\sigma_{\alpha\beta}(\omega)$ and the Seebeck tensor $S_{\alpha\beta}$, is outlined in three steps. First, we calculate the band structure for different stacking configurations of bilayer graphene ranging from AB-stacking, to the saddle-point (SP) configuration, and to the BA-stacking. Each of these configurations corresponds to a different displacement vector describing the relative lateral displacement between the two graphene layers, as depicted in a Supplementary Fig. 4 of a shear-type domain wall. Second, once the band structures are known, we calculate $\sigma_{\alpha\beta}(\omega)$ and $S_{\alpha\beta}$ for each of the stacking configurations. Finally, we define the spatial profile of the displacement vector in mTBG, yielding the spatial profile of $\sigma_{\alpha\beta}(\omega)$ and $S_{\alpha\beta}$.

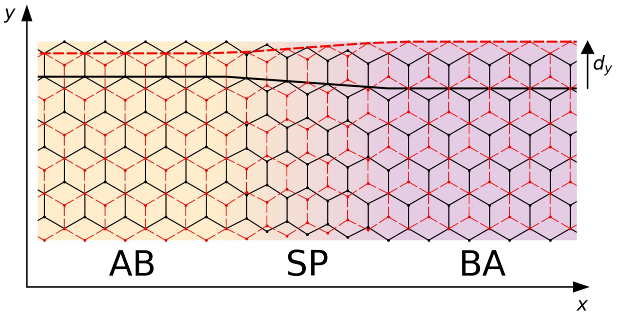

Supplementary Figure 4 | Local stacking configuration for a shear-type domain wall. The displacement d_y increases by a_0 when translating from an AB region to a BA region, while crossing the saddle-point configuration.

Band structure for bilayer graphene. We calculate the electronic band structure of bilayer graphene with an arbitrary displacement between the two layers. Using the model in Ref. 8, we define the displacement vector $d_y\mathbf{e}_y$ along the y-direction. With d_y defined in units of nearest-neighbour distance $a_0 = 0.142$ nm, $d_y = 1, 1.5, 2$ correspond to AB, SP, BA stacking configurations respectively.

For each stacking configuration between the AB and BA sites, we consider an infinite lattice of that configuration, and solve the eigenvalue problem

$$\mathcal{H}(\mathbf{k})u_\nu(\mathbf{k}) = \epsilon_\nu(\mathbf{k})u_\nu(\mathbf{k}), \tag{S16}$$

with the effective 4×4 Hamiltonian matrix given by

$$\mathcal{H}(\mathbf{k}) = \begin{bmatrix} H_0^+ & U^\dagger \\ U & H_0^- \end{bmatrix}, \tag{S17}$$

in which H_0 is the Hamiltonian of single layer graphene (SLG), and U describes the interaction potential between the two layers

$$H_0^\pm(\mathbf{k}) = \begin{bmatrix} \pm\Delta/2 & \hbar v(\xi k_x + ik_y) \\ \hbar v(\xi k_x - ik_y) & \mp\Delta/2 \end{bmatrix}, \tag{S18}$$

$$U = \frac{\gamma_1}{3}\left(1 + 2\begin{bmatrix} \cos\left(\frac{2\pi}{3}d_y\right) & \cos\left(\frac{2\pi}{3}(d_y+1)\right) \\ \cos\left(\frac{2\pi}{3}(d_y-1)\right) & \cos\left(\frac{2\pi}{3}d_y\right) \end{bmatrix}\right). \tag{S19}$$

Here Δ is the interlayer potential energy at each of the two layers, $v \approx 1 \times 10^6$ m/s is the band velocity of SLG, $\xi = \pm 1$ selects between the K and K' valley and $\gamma_1 \approx 0.4$ eV is the interlayer coupling. Since we change the carrier density in mTBG solely with one gate, the chemical potential μ is always

positioned outside of the displacement field induced bandgap, and therefore any corrections to this model due to interface states[12,13] are beyond the scope of this work.

In our model we assume the applied back gate voltage fixes the carrier density n everywhere in our mTBG device, and that the chemical potential $\mu(d_{\mathrm{y}}, n)$ varies spatially due to the varying density of states found for different stacking configurations present in the moire lattice of mTBG. To calculate the chemical potential, we first fix the interlayer potential through a simple capacitance model for bilayer graphene above a backgate. In this model $n(\Delta) = \frac{2\epsilon_0}{e^2 d_0}\Delta$, where $d_0 = 0.34$ nm corresponds to the vacuum distance between two layers. Knowing the density of states for every stacking configuration and calculated interlayer potential, we then build a spatial profile of the spatially varying chemical potential in our devices $\mu(d_{\mathrm{y}}, n)$.

Calculation of the Seebeck and conductivity tensors. Following the same definitions as in Ref. 14, we calculate for each stacking configuration the Seebeck tensor $S_{\alpha\beta}$ and the optical conductivity $\sigma_{\alpha\beta}(\omega)$. These tensors relate respectively the heat and current responses in the α direction under an applied electric field in the β direction. The Seebeck tensor is defined under the Relaxation Time Approximation as

$$S_{\alpha\beta} = -\frac{1}{eT}\frac{\mathcal{W}^{(1)}_{\alpha\beta}}{\mathcal{W}^{(0)}_{\alpha\beta}}, \quad \text{(S20)}$$

with

$$\mathcal{W}^{(p)}_{\alpha\beta} \equiv -\pi g \sum_\nu \int \frac{\mathrm{d}^2\mathbf{k}}{(2\pi)^2} f'_{\mathbf{k}\nu} \cdot (\epsilon_{\mathbf{k}\nu} - \mu)^p \langle u_{\mathbf{k}\nu}|\partial_{k_\alpha}\hat{H}(\mathbf{k})|u_{\mathbf{k}\nu}\rangle \langle u_{\mathbf{k}\nu}|\partial_{k_\beta}\hat{H}(\mathbf{k})|u_{\mathbf{k}\nu}\rangle. \quad \text{(S21)}$$

Here, $g = 4$ corresponds to the fourfold valley/spin degeneracy, f' is the derivative of the Fermi-Dirac distribution f. Furthermore, ν counts over the four bands and ∂_{k_j} is the momentum derivative in the direction j. For the electrical conductivity we used the Kubo formula[14] with an energy broadening $\eta = 10$ meV.

Profile of the displacement vector. Previous work[7] has experimentally determined the profile of the displacement vector $d_{\mathrm{y}}(x_{\mathrm{d}})$ for a distance x_{d} to the middle of a domain wall. This profile can be described as

$$d_{\mathrm{y}}(x_{\mathrm{d}}) = 1 + \frac{2}{\pi}\arctan\left(e^{\pi x_{\mathrm{d}}/l_{\mathrm{DW}}}\right), \quad \text{(S22)}$$

with $l_{\mathrm{DW}} = 6.2$ nm encoding the width of a shear domain wall, and $l_{\mathrm{DW}} = 10.1$ nm corresponding to the width of the energetically less-favoured tensile domain wall.

By combining the definitions in this section, we obtain a spatial map of $\sigma_{\alpha\beta}(\omega)$ and $S_{\alpha\beta}$ in a network of domain walls, by evaluating at each position the distance x_{d} to the nearest domain.

Supplementary Note 7: Impact of strain inside the AB domains

As mentioned in the main text, the spatially moving zero-crossing observed in the gate-voltage response (dotted line in Fig. 2d of the main text) is not captured in our PTE model. To illustrate this, Supplementary Fig. 5a shows our simulation of the gate-voltage response presented in the main text and Supplementary Note 4. It plots the simulated photovoltage for various carrier concentrations along the line trace that represents the experimental data of Fig. 2d of the main text. In comparison with the experimental data, our model accounts for two sign changes: one at charge neutrality (CNP, $n = 0$), and the other away from CNP and depending on the position (x_{tip}) within the moiré domain. The sign reversal at CNP is caused by the sign-reversal of the Seebeck coefficient with charge polarity, whereas the spatially dependent sign change away from CNP within the domains is a result of a competition of opposing photoresponse from opposite domain walls. Since the generated photovoltage is antisymmetric with respect to the domain wall (see Fig. 2c of the main text), positions of zero-crossings in the spatial profile of the photovoltage map depend strongly on the alignment of nearby domains to the projection of current flows (Fig. 3f,g of the main text). Despite this complex interplay, the spatial Seebeck profile in our model does not reveal a spatially dependence of the photovoltage sign change away from CNP.

To obtain a better understanding of the possible origins of the spatially dependent sign change, we explore two modifications to the Seebeck coefficient used in our current model and their impact on the calculated photovoltage maps. The first modification consists of a spatially varying Seebeck coefficient in the moiré unit cell, for example, due to strain[15]. Supplementary Fig. 5b plots an example of a spatially varying Seebeck coefficient, similar to the one presented in the main text, but modelled to be ∼1.5% lower in the central part of a domain. This profile essentially describes the case where the AB/BA stacked regions are more strained close to edges of the domains, which seems reasonable considering the large strain distributions found close to domain walls[7,16]. Considering a 1D system, this particular modification will generate a photoresponse that has an antisymmetric profile between two domains (see Eq. (2) of the main text), but with an opposite sign as compared to the photoresponse generated by the domain walls themselves. The second modification we consider is an enhancement of the Seebeck coefficient at the domain walls (see Supplementary Fig. 5b,c). The reason for this is two-fold. First, we note that our originally calculated Seebeck coefficient at the domain wall (green dashed line in Supplementary Fig. 5c, corresponding to a SP site) is significantly smaller than that at the middle of the domain (green solid line in Supplementary Fig. 5c, corresponding to an AB site). Because the difference between Seebeck coefficients at the SP and AB sites remains large for all doping levels in our model, the photoresponse also remains strong away from CNP (Supplementary Fig. 5a). However, our measured photovoltage instead decays rapidly with increasing doping (Fig. 2e of the main text), suggesting the difference between Seebeck coefficients at the two sites should reduce with increasing carrier density. Second, a reduced photovoltage generation by the domain walls allows any other small photovoltage contribution to yield a measurable influence, for example, one arising from a spatially varying Seebeck coefficient in the AB stacked domains.

We employ a simple toy model to evaluate the effect of these modifications in our mTBG lattice, considering the same measurement configuration as in Fig. 2a of the main text. We approximate the lattice structure as a square lattice of domains, together with a domain running along the diagonal. However, since the current flow direction is parallel to the diagonal domain in this measurement configuration, we can consider its contribution to the measured photovoltage neglectable (Fig. 2a of the main text). Whilst the diagonal domain should in principle influence the strain profile, we neglect this additional complication to gain some intuition into the possible effects of strain. Therefore, we consider the spatial photoresponse for a 1D channel (Eq. (2) of the main text) that crosses two parallel

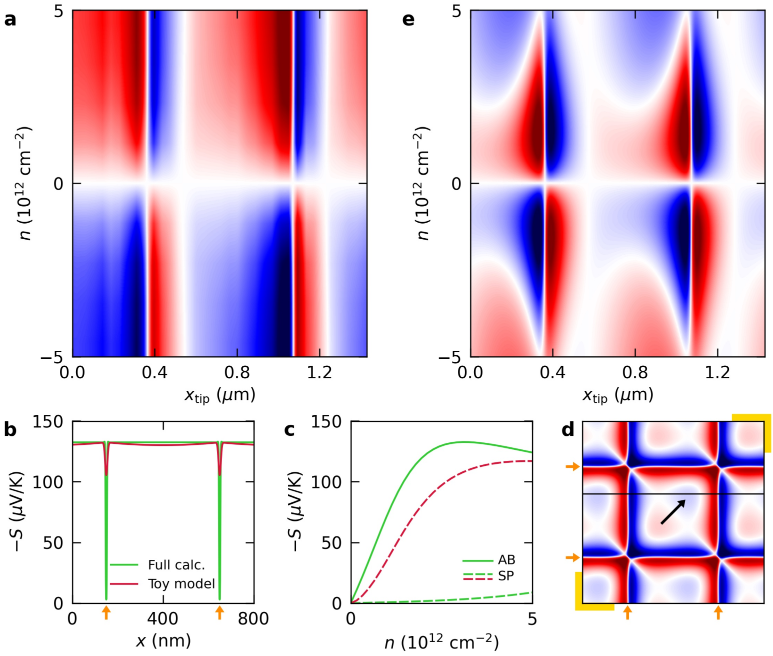

Supplementary Figure 5 | Additional contribution to the Seebeck profile due to strain. a Plot of the simulated photovoltage along the same line cut as in the experimental data (Fig. 2**d** of the main text) for a range of carrier densities (n). The simulated V_{PV} is calculated using the same method as presented in Supplementary Note 4. Length of scale bar: 500 nm; colour code: blue: -1, red: +1. **b** Calculated Seebeck coefficient according to the full calculation (green) crossing two domain walls at n = 3 x 10^{12} cm^{-2}, serving as input for the photovoltage simulation shown in panel **a**. Our toy model (red) uses an enhanced Seebeck coefficient at the domain walls (position indicated by the orange arrows) and a slightly reduced Seebeck coefficient between the domains. **c** Carrier density dependence of the Seebeck coefficient at the AB and SP sites (corresponding to the middle of the domain and domain wall, respectively) for both models, highlighting the enhanced Seebeck coefficient at the SP in our toy model. **d** Spatial profile of the calculated photovoltage in our toy model with n = 3 x 10^{12} cm^{-2} with the position of domain walls indicated by the orange arrows. The black arrow marks the current flow direction, with the position of the contacts indicated by the gold corners. **e** Line trace of the calculated photovoltage based on our toy model as function of carrier density. The line trace is taken along the black line in panel **d**.

domains. Since the current flow direction is aligned to the diagonal, both the horizontal and vertical domains contribute equally to the total photovoltage and thus we can simply sum up both contributions to build a spatial map.

Supplementary Figure 5d shows such a modelled spatial map for a fixed n = 3 x 10^{12} cm^{-2}, which highlights the interplay of various photovoltage contributions. In particular, two patches of photoresponse emerge with opposite sign inside the moiré domains, and change polarity at CNP. Supplementary Fig. 5e plots the gate-voltage response calculated in our toy model, taken across the line trace drawn in Supplementary Fig. 5d. Importantly, it contains the main features present in our full calculation (Supplementary Fig. 5a): sign reversals of the photoresponse at CNP and spatially in the domains. In addition, the details show some closer resemblance to our experimental data (Fig. 2d of the main text). In this toy model, the photovoltage from the domain walls reduces towards higher carrier densities, allowing the contribution due to the spatially-varying Seebeck coefficient to dominate. The competition between these two contributions causes the sign-change (away from CNP) to vary spatially as observed in our experimental data (Fig. 2d of the main text). This simple toy model illustrates qualitatively the possible influence of strain and enhanced Seebeck coefficients at the domain walls in influencing the photoresponse of our mTBG devices, even though the exact two-dimensional strain profile, including the influence of the diagonal domains has been ignored.

Supplementary Note 8: Cooling length in our devices

The photoresponse generated by the photo thermoelectric effect is driven by local temperature gradients generated in the electron gas in the vicinity of inhomogeneities in the Seebeck coefficient, which decay over a characteristic length scale from the source. Microscopically, this corresponds to the distance over which initial photoexcited carriers equilibrate with the lattice and is referred to as the cooling length L_{cool}. In the PTE, a photocurrent can be generated as long as photoexcitation occurs within a typical distance L_{cool} from any junction that exhibits gradients in the Seebeck coefficient. In Bernal stacked bilayer graphene, the cooling length has been measured to be around ~250 nm[17]. This is why in our mTBG samples the spatial photocurrent profile is so complex, because thermal gradients generated by photoexcitation in the middle of moiré domains can reach different surrounding junctions, which add linearly and contribute to the globally measured photoresponse. As mentioned in the main text, our samples tend to have extrinsic junctions in the form of stacking faults that also generate photocurrent and, in the case that they are located a distance L_{cool} from the superlattice region, would contribute a background signal to the photoresponse measured in our moiré domains. They could explain, for example, the constant negative photoresponse observed in the moiré domains at high doping levels (Fig. 2d and Fig. 4a of the main text). To rule out such contributions and allow correct interpretation of the photoresponse from moiré domains alone, we studied how the photoresponse from stacking faults behaves and measure L_{cool} in our devices.

Supplementary Fig. 6a plots an extended photocurrent map of the device presented in the main text (Fig. 4a of the main text) at a carrier density $n \sim 4 \times 10^{12}$ cm^{-2}, that includes the single layer graphene (SLG) region. The map clearly shows photocurrent hot spots on one side of the device originating from SLG-mTBG interface (marked by green dotted line), and on the other side from cracks/stacking faults (black dotted line). In between these interfaces we observe the anomalous negative photoresponse in the moiré domains. However, the photocurrent hotspots can be seen to decay around 1 μm into the sample, suggesting another possible origin to the photoresponse observed in the moiré domains. To evaluate this, we extracted L_{cool} from the SLG-mTBG interface. Supplementary Fig. 6b plots a line trace taken across the SLG-mTBG interface that extends a few microns into the mTBG region. Since the photocurrent profile is locally invariant under translations along the SLG-mTBG interface, we use the 1D version of our model of the PTE (Eq. (2) of the main text). This simplifies the analysis, as the

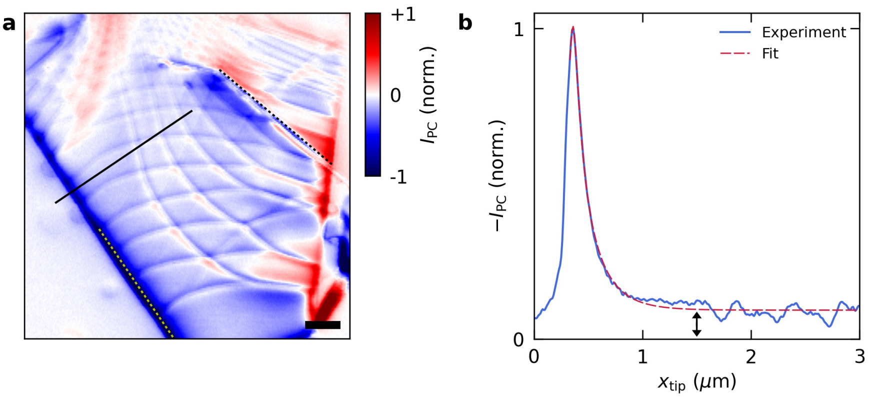

Supplementary Figure 6 | Extraction of cooling length. a Extended photocurrent map of Fig. 4a of the main text (E = 117 meV, $n \sim 4 \times 10^{12}$ cm^{-2}). The yellow dotted line marks partially marks the interface between SLG on the left side and mTBG on the right side, while the black dotted line marks a crack/stacking fault in our device. Length of scale bar: 500 nm. **b** Line trace of the photocurrent taken along the black line in panel **a**. The peak in photocurrent marks the position of the SLG-mTBG interface. A fit of the photocurrent profile yields a cooling length of 240 nm, and a background offset whose magnitude is a fraction ~0.1 of the photocurrent generated from the SLG-mTBG interface.

photocurrent on either side of the interface is simply proportional to increase in electron temperature δT (ignoring the domain walls further away) caused by local heating. That said, we should consider that heat spreads radially away from the excitation position, according to the following profile[18]

$$\delta T(x) \propto K_0\left(\sqrt{\left(\left(x - x_{\mathrm{tip}}\right)^2 + L_{\mathrm{tip}}^2\right)/L_{\mathrm{cool}}^2}\right), \tag{S23}$$

where K_0 is the modified Bessel function of the second kind, L_{tip} corrects for the finite radius of the AFM tip, and L_{cool} is the cooling length.

Importantly, we find Eq. (S23) alone does not describe our experimental data (Supplementary Fig. 6b). Instead, a constant offset is needed in our fit of Eq. (S23) (see arrow in Supplementary Fig. 6b) to describe the decay of photocurrent from the SLG-mTBG interface. From the decay of the photocurrent we extract L_{cool} = 240 nm. This behaviour, in addition to the constant off-set needed to describe out experimental data, shows that photocurrent generation from the SLG-mTBG is not responsible for the anomalous photocurrent observed at high doping in our devices (Fig. 4a of the main text).

Supplementary Note 9: Effects beyond the photo-thermoelectric effect

All our experimental evidence points towards an explanation of the observed photoresponse data based on the PTE. Indeed, the FEM simulations that include only this effect are in good agreement with experimental data and small discrepancies can be explained by a more complicated Seebeck coefficient profile as suggested by the simplified model in Supplementary Note 7. However, even if the general picture is well described by PTE, we cannot completely exclude that other effects contribute small additional corrections.

Here, we comment briefly on two other possible mechanisms of photoresponse generation, namely the photogalvanic effect and the photovoltaic effect. The photogalvanic effect generates a photocurrent thanks to the intrinsic *second-order* response of the material to the incident electric field that gives rise to a DC current density $\mathbf{j}_{\mathrm{PG}}(\mathbf{r}, \mathbf{r}_{\mathrm{tip}}) \propto |\mathbf{E}(\mathbf{r}, \mathbf{r}_{\mathrm{tip}}, \omega_{\mathrm{ph}})|^2$. Such a response is symmetry-forbidden for homogeneous materials (for unpolarised light) but can play a role in the presence of strong electronic density gradients or strain.

This modifies our Equation (S1) into

$$\mathbf{J}(\mathbf{r}) = -\sigma(\mathbf{r})\nabla V(\mathbf{r}) - \sigma(\mathbf{r})S(\mathbf{r})\nabla\delta T(\mathbf{r}) + \mathbf{j}_{\mathrm{PG}}(\mathbf{r}, \mathbf{r}_{\mathrm{tip}}), \quad \text{(S24)}$$

adding another source term.

We can write a relation similar to Eq. (1) of the main text for the PGE that reads

$$V_{\mathrm{PGE}}^{(m)} = \int d\mathbf{r}\, \boldsymbol{\mathcal{R}}_{\mathrm{PGE}}^{(m)}(\mathbf{r}) \cdot \mathbf{j}_{\mathrm{PG}}(\mathbf{r}, \mathbf{r}_{\mathrm{tip}}). \quad \text{(S25)}$$

Again, $\boldsymbol{\mathcal{R}}_{\mathrm{PGE}}^{(m)}(\mathbf{r})$ can be calculated by solving the thermoelectric transport equations using FEM and taking advantage of the Shockley-Ramo theorem[5,6,19], $\boldsymbol{\mathcal{R}}_{\mathrm{PGE}}^{(m)}(\mathbf{r})$ is in fact proportional to the gradient of the potential that would be present in the system in absence of sources and biasing the *m*-th with a constant current. Qualitatively, $\boldsymbol{\mathcal{R}}_{\mathrm{PGE}}^{(m)}(\mathbf{r})$ is a smooth vector field flowing mainly in the direction connecting the contacts at which photocurrent is measured. Importantly it is weakly affected by the cooling length. As a consequence, we expect PG features (if any) to be sharper with respect to PTE features that are smoothed on the length scale L_{cool}.

We note also that the photovoltaic effect can also play a role in the photoresponse of systems with more than one energy band. Including this effect in our model would require studying a system of three coupled equations including the imbalance density (electron density + holes density) and current. While this is outside the scope of this work, we tend to exclude this explanation since photovoltaic contributions should display a threshold behaviour as a function of the photon energy that is not observed in our experimental data.

Supplementary Note 10: Heating from hyperbolic phonon polaritons

When the excitation energy lies within the reststrahlen band of hBN the optical response is dominated by the hyperbolic phonon polaritons (HPP) of the hBN. The wavevectors of the possible eigenmodes in an hBN slab is given by $k_n(\omega) = k_0(\omega) + n\,\Delta k(\omega)$, with k_0 representing the wavevector of the zeroth-order mode typically observed in s-SNOM experiments[20,21], followed by equidistant modes separated by Δk, n being an integer. The presence of bilayer graphene minimally affects this spectrum, the main consequence being an additional damping of the modes due to graphene absorption, as we checked numerically using transfer-matrix method[22].

The s-SNOM tip launches in general a linear combination of these modes (like a ray). Ignoring losses and considering for the sake of simplicity a 1D problem ($q_y = 0$), this combination can be written as a Bloch wave $E_x(x,z) = e^{ik_0(x-x_{\text{tip}})}u(x - x_{\text{tip}}, z)$, where u is a periodic function of x with periodicity $\lambda_{\text{ray}} \equiv 2\pi/\Delta k$. The periodicity follows from the constant spacing in momentum space of the modes. Taking into account the y dimension and losses will introduce a geometrical $1/\sqrt{r}$ attenuation and an exponential damping but will not alter the general picture.

An important question is whether it is more correct to compare the distance d (as determined in Fig. 4c of the main text) with $2\pi/k_0$ (representing the fundamental eigenmode) or with $2\pi/\Delta k$ (representing the ray-like mode). When measuring an interference pattern from reflected HPPs[20,21,23], it is correct to use $2\pi/k_0$ since the phase $e^{ik_0(x-x_{\text{tip}})}$ is the most important factor in determining the interference. However, our photocurrent generation mechanism is based on the amount of heat that is locally injected into the bilayer graphene. As heating is an incoherent mechanism, it is only sensitive to the intensity pattern encoded in $u(x - x_{\text{tip}})$ while being insensitive to the phase.

In particular, the periodicity of $u(x - x_{\text{tip}})$ creates copies of the field hot-spot generated by the tip at distances multiples of λ_{ray}. When one of these copies (the first one) comes close to the domain wall (on the opposite side with respect to the tip) it creates a photocurrent that partially counteracts the one created by the original tip hot-spot, leading to a reduction of the signal. We therefore fitted d (see Fig. 4d of the main text) with $d(\omega) = a\lambda_{\text{ray}}(\omega)$.

The length λ_{ray} has an intuitive geometrical interpretation in terms of rays travelling at a fixed angle θ_{BN} with respect to the anisotropic axis z of the hBN[24]. This allows us to express the in-plane phonon-ray wavelength as $\lambda_{\text{ray}} = 2t \cdot \tan(\theta_{\text{BN}})$ where t is the total thickness of the hBN layers, and

$$\theta_{\text{BN}} = \tan^{-1}\left[\text{Re}\left(\frac{i\sqrt{\epsilon_{\text{x,y}}}}{\sqrt{\epsilon_{\text{z}}}}\right)\right]$$

determined by the dielectric function ϵ of hBN (in all the calculations we used the model of the hBN dielectric function from Ref. 24).

Interference between the HPPs launched by the tip and waves reflected from the domain wall could in principle modify the heating pattern by producing a reflected Bloch wave $R \cdot e^{-ik_0 x}\,\tilde{u}(x,z)$, ($\tilde{u}(x,z)$ is not in general equal to $u(x,z)$) and therefore adding a component of the injected power with spatial frequency $2k_0$. We expect, however, this effect to be a minor correction with respect to the intensity modulation encoded in $u(x - x_{\text{tip}})$ inside the reststrahlen band. As we checked numerically[22], the spectrum of HPPs is almost unaffected even when we remove completely the graphene sheet. This means that even a large modification of the optical response of graphene is likely to produce only a small reflected wave. Note that this does not conflict with the observation of reflection of HPPs at domain walls reported in scattering near field experiments[23]. Since the contrast in scattering

experiments is *only* due to interference with the reflected waves even a small reflected wave ($|R| \ll 1$) can produce measurable effects.

The situation is different outside the reststrahlen band when the periodic structure due to $u(x - x_{\text{tip}})$ is not present anymore and interference between launched and reflected waves can lead to the formation of interferences fringes. Previous s-SNOM studies have shown bright features due to reflected polaritons by domain walls[25,26]. Therefore, we think that the fringes observed in our data outside the hBN reststrahlen band (Fig. 1d, Fig. 2d and Fig. 4a of the main text) are the result of plasmon polaritons reflection.

Supplementary References

1. Woessner, A. *et al.* Highly confined low-loss plasmons in graphene–boron nitride heterostructures. *Nat. Mater.* **14**, 421–425 (2015).
2. Lundeberg, M. B. *et al.* Thermoelectric detection and imaging of propagating graphene plasmons. *Nat. Mater.* **16**, 204–207 (2017).
3. Alonso-González, P. *et al.* Acoustic terahertz graphene plasmons revealed by photocurrent nanoscopy. *Nat. Nanotechnol.* **12**, 31–35 (2017).
4. Ju, L. *et al.* Photoinduced doping in heterostructures of graphene and boron nitride. *Nat. Nanotechnol.* **9**, 348–352 (2014).
5. Torre, I. Diffusive solver: a diffusion-equations solver based on FEniCS. *arXiv:2011.04351* (2020).
6. Lundeberg, M. B. & Koppens, F. H. L. Thermodynamic reciprocity in scanning photocurrent maps. *arXiv:2011.04311* (2020).
7. Alden, J. S. *et al.* Strain solitons and topological defects in bilayer graphene. *Proc. Natl. Acad. Sci.* **110**, 11256–11260 (2013).
8. Koshino, M. Electronic transmission through AB-BA domain boundary in bilayer graphene. *Phys. Rev. B* **88**, 115409 (2013).
9. Dean, C. R. *et al.* Boron nitride substrates for high-quality graphene electronics. *Nat. Nanotechnol.* **5**, 722–726 (2010).
10. Bandurin, D. A. *et al.* Negative local resistance caused by viscous electron backflow in graphene. *Science* **351**, 1055–1058 (2016).
11. Tielrooij, K. J. *et al.* Out-of-plane heat transfer in van der Waals stacks through electron-hyperbolic phonon coupling. *Nat. Nanotechnol.* **13**, 41–46 (2018).
12. Ju, L. *et al.* Topological valley transport at bilayer graphene domain walls. *Nature* **520**, 650–655 (2015).
13. Yin, L.-J., Jiang, H., Qiao, J.-B. & He, L. Direct imaging of topological edge states at a bilayer graphene domain wall. *Nat. Commun.* **7**, 11760 (2016).
14. Novelli, P., Torre, I., Koppens, F. H. L., Taddei, F. & Polini, M. Optical and plasmonic properties of twisted bilayer graphene: Impact of interlayer tunneling asymmetry and ground-state charge inhomogeneity. *Phys. Rev. B* **102**, 125403 (2020).
15. Nguyen, M. C., Nguyen, V. H., Nguyen, H.-V. V., Saint-Martin, J. & Dollfus, P. Enhanced Seebeck effect in graphene devices by strain and doping engineering. *Phys. E Low-dimensional Syst. Nanostructures* **73**, 207–212 (2015).
16. Kazmierczak, N. P. *et al.* Strain fields in twisted bilayer graphene. *arXiv:2008.09761* (2020).
17. Gabor, N. M. *et al.* Hot carrier-assisted intrinsic photoresponse in graphene. *Science* **334**, 648–52 (2011).
18. Woessner, A. *et al.* Near-field photocurrent nanoscopy on bare and encapsulated graphene. *Nat. Commun.* **7**, 10783 (2016).
19. Song, J. C. W. & Levitov, L. S. Shockley-Ramo theorem and long-range photocurrent response in gapless materials. *Phys. Rev. B* **90**, 075415 (2014).
20. Dai, S. *et al.* Tunable Phonon Polaritons in Atomically Thin van der Waals Crystals of Boron Nitride. *Science* **343**, 1125–1129 (2014).
21. Dai, S. *et al.* Subdiffractional focusing and guiding of polaritonic rays in a natural hyperbolic material. *Nat. Commun.* **6**, 6963 (2015).

22. Woessner, A. *et al.* Highly confined low-loss plasmons in graphene–boron nitride heterostructures. *Nat. Mater.* **14**, 421–425 (2015).

23. Luo, Y. *et al.* In situ nanoscale imaging of moiré superlattices in twisted van der Waals heterostructures. *Nat. Commun.* **11**, 4209 (2020).

24. Caldwell, J. D. *et al.* Sub-diffractional volume-confined polaritons in the natural hyperbolic material hexagonal boron nitride. *Nat. Commun.* **5**, 5221 (2014).

25. Jiang, L. *et al.* Soliton-dependent plasmon reflection at bilayer graphene domain walls. *Nat. Mater.* **15**, 840–844 (2016).

26. Jiang, B.-Y. *et al.* Plasmon Reflections by Topological Electronic Boundaries in Bilayer Graphene. *Nano Lett.* **17**, 7080–7085 (2017).
